# Testing for intraspecific postzygotic isolation between cryptic lineages of *Pseudacris crucifer*

**DOI:** 10.1002/ece3.851

**Published:** 2013-10-23

**Authors:** Kathryn A Stewart, Stephen C Lougheed

**Affiliations:** Department of Biology, Queen's UniversityKingston, Ontario, Canada, K7L 3N6

**Keywords:** cryptic diversity, hybridization, intraspecific divergence, reproductive isolation

## Abstract

Phenotypically cryptic lineages appear common in nature, yet little is known about the mechanisms that initiate and/or maintain barriers to gene flow, or how secondary contact between them might influence evolutionary trajectories. The consequences of such contact between diverging lineages depend on hybrid fitness, highlighting the potential for postzygotic isolating barriers to play a role in the origins of biological species. Previous research shows that two cryptic, deeply diverged intraspecific mitochondrial lineages of a North American chorus frog, the spring peeper (*Pseudacris crucifer*), meet in secondary contact in Southwestern Ontario, Canada. Our study quantified hatching success, tadpole survival, size at metamorphosis, and development time for experimentally generated pure lineage and hybrid tadpoles. Results suggest that lineages differ in tadpole survival and that F_1_ hybrids may have equal fitness and higher than average mass at metamorphosis compared with pure parental crosses. These findings imply hybrid early life viability may not be the pivotal reproductive isolation barrier helping to maintain lineage boundaries. However, we observed instances of tadpole gigantism, failure to metamorphose, and bent tails in some tadpoles from hybrid families. We also speculate and provide some evidence that apparent advantages or similarities of hybrids compared with pure lineage tadpoles may disappear when tadpoles are raised with competitors of different genetic makeup. This pilot study implies that ecological context and consideration of extrinsic factors may be a key to revealing mechanisms causing negative hybrid fitness during early life stages, a provocative avenue for future investigations on barriers to gene flow among these intraspecific lineages.

## Introduction

Many studies have revealed cryptic species with little to no obvious morphological differentiation, but levels of neutral genetic divergence equal to or greater than those between morphologically diagnosable species (Avise 2000). Although such cryptic species are commonly reported in tropical taxa (Elmer et al. 2007; Fouquet et al. 2007), fewer have been reported in North American phylogeographical and phylogenetic surveys, although there are some striking examples (Highton 1995; Zamudio and Savage 2003) including frogs (e.g., in *Pseudacris*; Lemmon et al. 2008). Some studies reveal that these phenotypically cryptic lineages may have substantial barriers to gene flow, and in some cases have been evolving independently for millions of years (Phillips et al. 2004; Hoskin et al. 2005; Fouquet et al. 2007; Kawakami et al. 2009; Singhal and Moritz 2012; Elmer et al. 2013). Without overt phenotypic differentiation however, important evolutionary questions remain regarding mechanisms that maintain lineage boundaries or the nature of reproductive isolation (Bickford et al. 2006; Singhal and Moritz 2012). Indeed, some ask whether these cryptic lineages are nascent or fully independent species, or simply “evolutionary ephemera” (Avise and Wollenberg 1997).

Biological species are defined by reproductive isolation barriers – the suite of mechanisms (genetic, physiological, or behavioral) that maintain distinct gene pools by preventing the production of viable or fertile offspring (Mayr 1963). Thus, understanding biological speciation requires that we examine factors associated with the evolution of pre- and postzygotic barriers among natural populations. Most animal sister species begin as geographically isolated conspecific populations (Barraclough and Vogler 2000; Coyne and Orr 2004; Fitzpatrick et al. 2009), with secondary contact between diverging populations potentially being important in shaping evolutionary trajectories (Servedio 2001; Martin et al. 2010). Reproductive barriers evolved in allopatry are not always impermeable and may allow gene flow between lineages upon secondary contact, with various outcomes including (1) fusion or genetic assimilation where one population predominates (Arnold and Hodges 1995; Burke and Arnold 2001), (2) formation of a stable “hybrid swarm” (Nielsen et al. 2003; Seehausen et al. 2003), or (3) reinforcement of species boundaries should maladaptive hybridization strengthen incipient species-recognition systems (Dobzhansky 1937; Blair 1955; Rundle 2002; Geyer and Palumbi 2003; Schluter 2003). Ultimately then, the outcome of secondary contact depends on hybrid fitness, highlighting the potential importance of postzygotic isolating barriers in the origins of biological species.

In the traditional allopatric model of speciation, geographically isolated populations gradually accumulate an array of mutations, be they beneficial, mildly deleterious, or neutral, as a correlated response to genetic divergence and time (Mayr 1942). Upon secondary contact, hybrids between these two diverging populations are less fit than their pure counterparts as a result of negative epistatic interactions, referred to as Dobzhansky–Muller Incompatibilities (DMI; Dobzhansky 1937; Muller 1940). Postzygotic incompatibilities may be intrinsic (inviability or sterility) or extrinsic (ecological or behavioral/sexual dysfunction), and the magnitude and direction of effects may occur differentially over the lifespan of an individual, perhaps being favorable at one life stage and detrimental in another (Parris 1999; Coyne and Orr 2004; Lemmon and Lemmon 2010). Complicating matters further, selection against hybridization is frequently not equal between populations and species, often apparent in asymmetrical fitness costs (Parris 1999; Pearson 2000; Tiffin et al. 2001; Veen et al. 2001; Pfennig and Simovich 2002). Moreover, hybrids are not always unfit (Arnold 1997), and there is growing evidence for the maintenance of stable hybrid zones (Slatkin 1973; Endler 1977; Barton 1979; Mallet 1986; Brelsford and Irwin 2009), hybrid superiority over parental individuals (May et al. 1975; Moore 1977), and even hybrid speciation (via allopolyploidy – Wood et al. 2009; Mable et al. 2011; homoploid hybrid speciation – Welch and Rieseberg 2002; Lai et al. 2006; Mallet 2007; Jiggins et al. 2008; Mavárez and Linares 2008; Hegarty et al. 2009; Hermansen et al. 2011), further highlighting the need for hybrid assessments.

**Figure 1:**
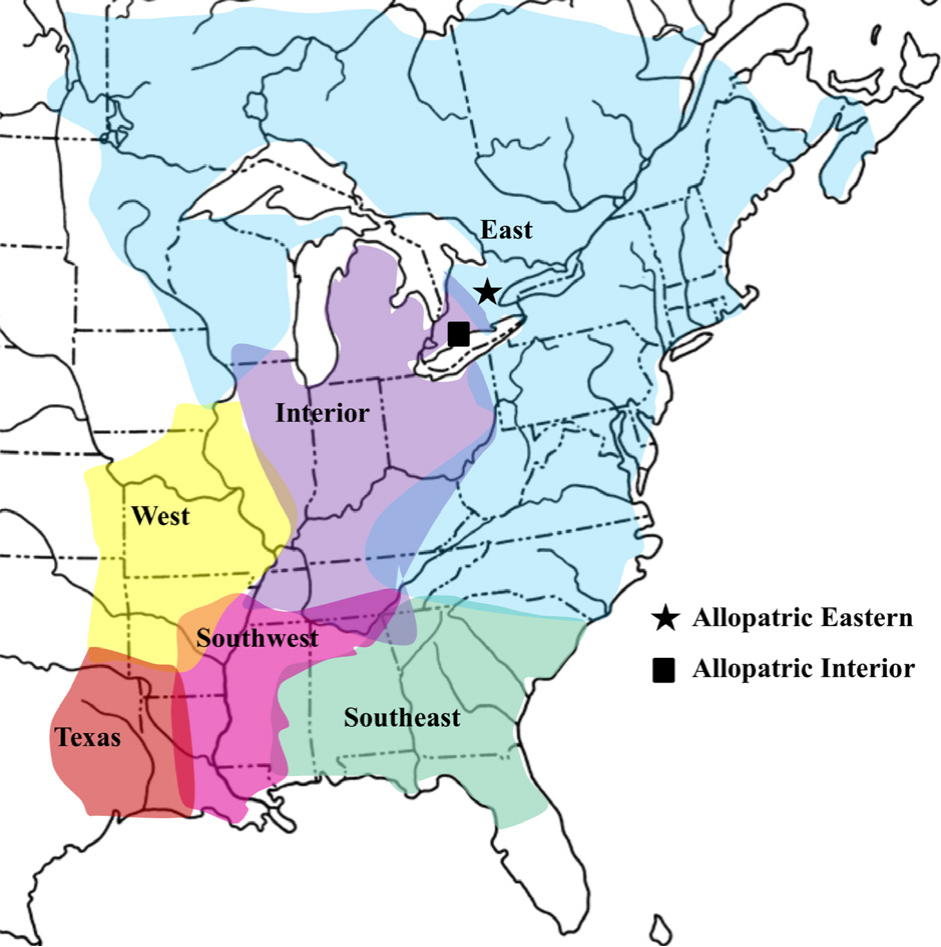
*Pseudacris crucifer* distribution and sampling map. Current range of the spring peeper (*P. crucifer*) in Eastern North America. Shown are the five mitochondrial lineages described by Austin et al. (2004), plus a more recently found lineage in eastern Texas (“Texas” lineage; Stewart 2013; J. D. Austin, unpubl. data). Sampled allopatric populations for Eastern (star) and Interior (square) lineage parental individuals used for hybrid crosses are depicted.

The spring peeper (*Pseudacris crucifer*), a North American chorus frog from the family Hylidae, is an excellent system for studying the consequences of secondary contact and hybridization on the evolution of reproductive isolation between cryptic lineages. Earlier work revealed a dynamic history of range fragmentation, range expansion, and secondary contact among mitochondrial lineages across the species range (Austin et al. 2002, 2004; see Fig. 1 for a distribution map). In Southwestern Ontario, Canada, two such lineages (designated Eastern and Interior; Fig. 1), which began diverging approximately 2.5–5 million years ago (Austin et al. 2002, 2004; Stewart 2013), now meet in secondary contact (Austin et al. 2002). Preliminary genetic evidence indicates natural hybridization between lineages with some phenotypic differences between parental allopatric populations in morphology and male advertisement call (Stewart 2013). However, fundamental to understanding the consequences of secondary contact are data on fitness costs of hybridization and thus possible postzygotic isolation barriers. Here, we conduct reciprocal hybrid crosses between spring peepers from these diverging lineages and quantify key components of hybrid fitness from hatching to metamorphosis.

Despite the deep dates of the onset of their divergence, secondary contact between these intraspecific lineages probably occurred at most 15,000 years ago following the most recent glacial retreat in the Northern Unites States and Southern Ontario (Austin et al. 2002). Our objective for this study was to test for postzygotic barriers between these two lineages by assaying tadpole F_1_ hybrid viability and developmental rate. As evidence from both mitochondrial and nuclear DNA markers implies that lineage population boundaries have been maintained throughout much of the spring peeper's range despite prolonged contact (Austin et al. 2002, 2004; Stewart 2013; K. A. Stewart, J. D. Austin, and S. C. Lougheed, unpubl. data), we hypothesize the existence of strong postzygotic isolation barriers in early life-history traits as expected because of DMI. We predict experimentally generated hybrids should have lower fitness manifested in diminished hatching success, overall survival, size at metamorphosis, and development rate, compared with their pure tadpole counterparts.

## Materials and Methods

### The spring peeper

The reproductive ecology of spring peepers is well characterized. Males can be found in aggregations (choruses) in the spring where they set up small territories and actively call to attract females. Reproductively receptive females enter choruses, choose a male by approaching him, touch him on the shoulder, and then permit amplexus (Badger and Netherton 2004). Females then swim with males attached and external fertilization ensues, with eggs deposited on submerged vegetation (Wright and Wright 1995).

### Population sampling

We hand-collected males and females on two consecutive nights (April 10 and April 11, 2011) from allopatric populations of the two focal lineages (Fig. 1), located at least 50 km from the zone of secondary contact (Austin et al. 2002), and transported them live to the Animal Care Facility at the University of Guelph. Only males actively advertising, and females either caught in amplexus and/or gravid (eggs visible within the abdominal cavity) were used to ensure reproductive receptivity. Source populations for pure Eastern and Interior allopatric individuals were diagnosed previously (Austin et al. 2002, 2004; Stewart 2013) to confirm genetic ancestry.

### Hybrid crosses

We conducted a fully reciprocal hybridization experiment by creating the following crosses: Eastern females and males, Interior females and males, Interior males and Eastern females, and Eastern males and Interior females. To minimize maternal affects, we used eggs from the same females for both pure and interlineage crosses and nested sires within dams (Parris 1999). For the pure Interior crosses, five Interior females were crossed with six Interior males. These same Interior females were crossed with six Eastern males to create one class of F_1_ hybrids with Interior haplotypes (hereafter Interior hybrids). For the pure Eastern crosses, six Eastern females were crossed with five Eastern males (1 male was used twice with different females). These same Eastern females were crossed with six Interior males (1 female was used twice with different males) to create a reciprocal F_1_ hybrid treatment with Eastern haplotypes (hereafter Eastern hybrids). In total, we produced six pure Interior, six Interior hybrid, six pure Eastern, and seven Eastern hybrid family crosses (replicated twice, see below) from a total of 1957 eggs.

Females were injected with Luteinizing Hormone-Releasing Hormone (Sigma-Aldrich, Oakville, ON, Canada), held at 4°C for 8 h, and then brought to room temperature to induce ovulation (Silla 2011). Sperm suspensions were made by macerating a testis from each male in approximately 10 mL of aged (dechlorinated) tap water (Parris 1999). Crosses were performed by stripping between 40 and 100 eggs from each female in sequence and immersing them in the sperm suspension of the relevant male in a petri dish. We allowed 10 min for fertilization, after which the sperm suspension was diluted, and eggs covered in more aged tap water. Each male was used for two crosses only. Use of left and right testes was randomized, as was order of egg stripping, and crosses (in terms of hybrid vs. pure). All fertilizations were performed within 3 h of each other, well within a time period known to produce equivalent levels of fertilization success in previous experiments involving *Lithobates blairi* and *Lithobates sphenocephala* (Parris 1999, 2004). Experiments were conducted at ambient temperature within the facility (23°C) consistent with standard conditions used in other studies (Gosner and Rosman 1960; Earl et al. 2012). All larvae hatched within 7 days at which point we calculated hatching success from total number of eggs for each cross. To maintain equivalent densities, upon hatching tadpoles were chosen haphazardly and transferred to larger glass containers of aged tap water (2 L) of between 12 and 14 siblings, with each cross type replicated twice. Container placement was randomized and exposed to a 16:8 h light cycle within the University of Guelph, Animal Care Facilities. Containers were randomly rearranged every 7 days to minimize spatial affects, such as positioning near light sources (UV irradiation may reduce hatching success and tadpole development; Blaustein et al. 1998, 2001). Tadpoles were fed ad libitum boiled lettuce, containers were siphoned to remove feces, and aged water was continually added to maintain a volume of 2 L per container. Once tadpoles started to develop legs (“froglets” Gosner Stage 31–39; Gosner 1960), containers were angled at 15° to allow for a resting surface and covered with mesh to preclude escape. Froglets no longer employing an aquatic feeding regime were fed wingless *Drosophila melanogaster*. Full metamorphosis was considered to have been achieved at Gosner Stage 45–46 (Gosner 1960), when the tail was fully absorbed, at which time froglets were removed from their containers, sacrificed by immersion in tricaine methanesulfonate (MSS222), measured, and genotyped (see below). Spring peeper tadpoles require between 90 and 100 days for metamorphosis (Minton 2001); we thus ended the experiment at 100 days posthatching at which time 98.4% of tadpoles had fully metamorphosed (see “Deformities and developmental aberrations” for further details).

### Genotyping tadpoles

Spring peeper adults and tadpoles from divergent lineages cannot be distinguished through external morphology alone. We thus collected individuals as they died or metamorphosed (sacrificed metamorphs as above) and placed them in 95% ethanol with individual tracking numbers and date for later genetic identification (Supporting Information, Methods, Table S1). Tadpole DNA was extracted using a QIAGEN DNeasy kit (Mississauga, ON, Canada) according to the manufacturer's protocol and sequenced for a 692-bp fragment of the mtDNA cytochrome *b* (cyt *b*) using primers MVZ-15L and MVZ-18H (Moritz et al. 1992) as described in Austin et al. (2002).

### Tadpole fitness measurements

We measured a suite of fitness correlates for each cross treatment (Interior, Eastern, Interior hybrids, Eastern hybrids). Hatching success was determined as the proportion of tadpoles hatched from total eggs added to a particular sperm suspension. Survival rate was the proportion of tadpoles that survived to metamorphosis relative to those that hatched. Mortality was calculated as the cumulative measure of tadpoles that died per family, per day. Upon metamorphosis, we measured snout–vent length (SVL ± 0.2 mm) using digital calipers and mass (±0.02 g) using a digital scale (TR-2102; Denver Instrument Co., Bohemia, NY). Cumulative averages were tallied for rate of metamorphosis posthatch date, and cumulative rate of mortality posthatch, per cross treatment.

### Statistical analysis

**Figure 2:**
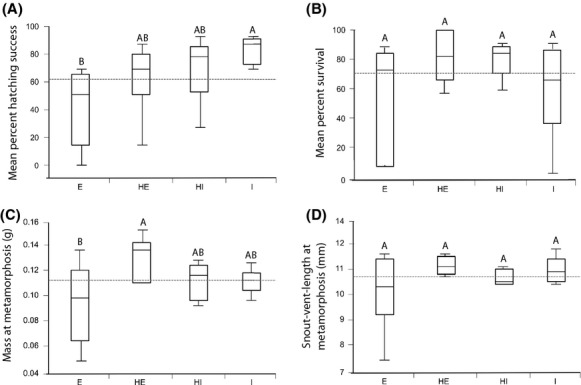
Tadpole fitness among genotypes. Boxplots with standard deviation bars for pure Eastern (E), hybrids with Eastern haplotypes (HE), hybrids with Interior haplotypes (HI) and pure Interior (I) tadpoles from in vitro crosses (*n* = 6, 7, 6, 6 crosses per treatment, respectively) (A) Mean hatching success (%), (B) Mean survival to metamorphosis (%), (C) Mass at metamorphosis (g), and (D) snout–vent length at metamorphosis (mm). Letters above boxplots denote significantly different means according to Tukey–Kramer HSD test. Dashed line represents grand mean across all families.

**Figure 3:**
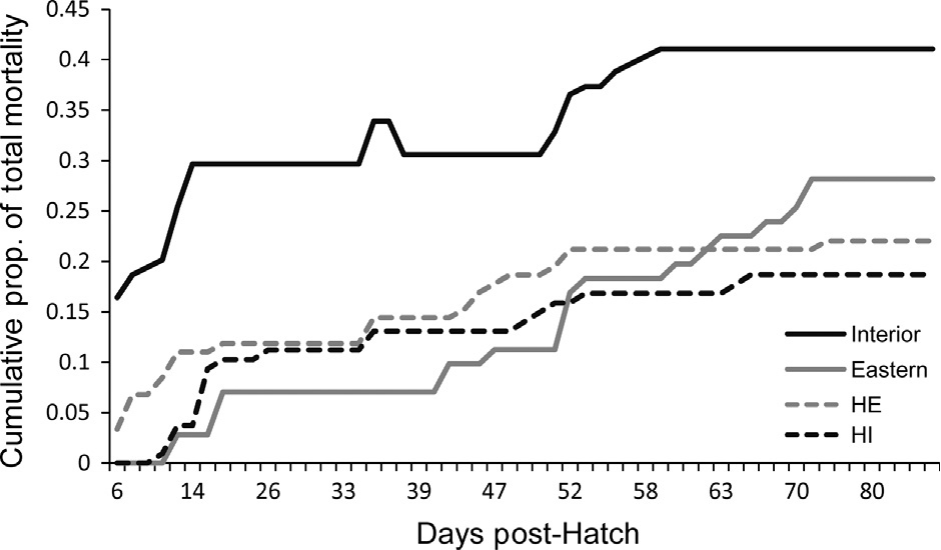
*Pseudacris crucifer* tadpole mortality. Cumulative proportion of total mortality for *P. crucifer* tadpoles hatched (day 0) tadpoles from different genetic lineages. Families were pooled by treatment types (*n* = 6, 6, 7, 6 for I, E, HE, and HI, respectively). Other details as in Figure 2.

**Figure 4:**
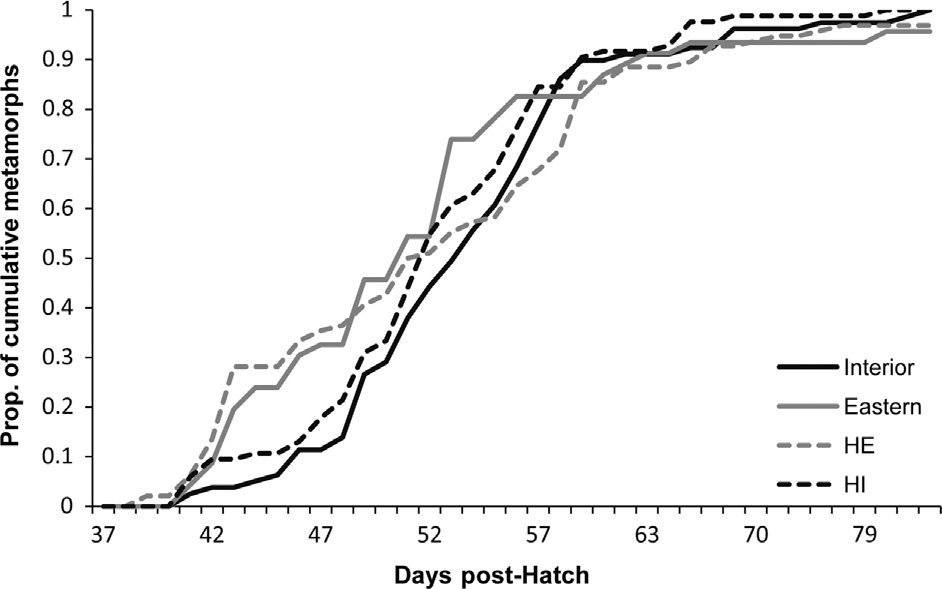
*Pseudacris crucifer* tadpole metamorphosis. Cumulative metamorphosis of spring peeper hatched (day 0) tadpoles from different cross treatments. Families were pooled by treatment type (*n* = 6, 6, 7, 6, for I, E, HE, and HI, respectively). Other details as in Figure 2.

To test for the effect of genetic ancestry on *P. crucifer* tadpole fitness, we compared percent hatching success (proportion of eggs hatched), percent survival to metamorphosis, mass (g) at metamorphosis, and size (SVL in mm) at metamorphosis, by performing one-way analyses of variance (ANOVA) and Tukey–Kramer HSD tests (also see Figs. 2–4 for details on mean differences among groups). Tadpole mortality and metamorphosis curves were compared using log-rank Mantel–Cox tests and displayed as cumulative measures over time. All analyses were conducted using JMP (version 10; SAS Institute Inc., Cary, NC).

## Results

### Tadpole fitness

For all crosses combined, we obtained a total of 1228 (62.75% of 1957) hatched eggs, all hatching occurring within 1 day among all crosses in all treatments. We found significant differences among treatments in tadpole hatching success (ANOVA *F*_3,25_ = 4.06, *P* = 0.0194; Fig. 2A). Tukey–Kramer HSD tests showed that success was significantly greater for pure Interior (Mean ± SE; 82.7% ± 3.87) compared with pure Eastern individuals (40.3% ± 10.23), with hybrids of both haplotypes displaying intermediate hatching success relative to their pure counterparts. One pure Eastern family cross completely failed and as such was not included in any other measure of tadpole fitness, reflected in diminished sample size for this treatment.

Mean survival was not significantly different among *P. crucifer* tadpoles (ANOVA *F*_3,24_ = 1.60, *P* = 0.22; Fig. 2B). Mortality curves, however, were significantly different among groups (log-rank χ^2^ = 14.24, df = 3, *P* = 0.0026; Fig. 3) with pure Interior tadpoles showing mortality earlier (Mean ± SE; 13.03 days ± 4.2) compared with Eastern tadpoles (53.1 days ± 5.6) in their developmental period, with both hybrid lines showing mortality curves similar to that of pure Eastern individuals. A Tukey–Kramer HSD test demonstrated mortality at 13 days posthatch to be significantly greater for pure Interior tadpoles than pure Eastern, with hybrids of both lineages showing similar rates of mortality to pure Eastern (ANOVA *F*_3,24_ = 283.1, *P* < 0.001).

We found no significant differences in time to metamorphosis: pure Eastern tadpoles (Mean ± SE; 49.34 days ± 3.8), pure Interior tadpoles (54.0 days ± 3.1; log-rank χ^2^ = 0.69, df = 3, *P* = 0.88; Fig. 4).

We found mass at metamorphosis to be significantly different among treatments (ANOVA *F*_3,23_ = 3.54, *P* = 0.033). Tukey–Kramer HSD tests showed that hybrids with Eastern haplotypes metamorphosed into adults at a significantly higher mass (Mean ± SE; 0.13 g ± 0.007) compared with pure Eastern (0.096 g ± 0.008), pure Interior (0.11 g ± 0.008), and hybrid individuals with Interior haplotypes (0.11 g ± 0.009; Fig. 2C). SVL at metamorphosis did not vary significantly among treatments (ANOVA *F*_3,23_ = 1.89, *P* = 0.164; Fig. 2D).

### Deformities and developmental aberrations

After 100 days posthatching, five tadpoles remained unmetamorphosed, four of which were hybrids with Eastern haplotypes and the other a pure Eastern tadpole (development remained stunted at Gosner Stage <30 with no limb development). Small sample sizes precluded formal statistical tests. These unmetamorphosed individuals exhibited gigantism, measuring 8–10 times the length of normally developing tadpoles at Gosner stage 30 (30 mm for unmetamorphosed tadpoles to 3–4 mm of normal tadpoles). Bent tails that caused tadpoles to swim in circles were also observed, ranging from 10.2% in hybrids with Eastern haplotypes to 3% in hybrids with Interior haplotypes, and 7% in pure Eastern individuals (pooled across families). Bent tails did not seem to impede metamorphosis or survival, but did compromise locomotion causing tadpoles to swim in circles.

## Discussion

Our study of tadpole fitness and the consequences of hybridization between two diverging intraspecific spring peeper lineages reveal some notable results: (1) we found marked differences between Eastern and Interior lineages in key aspects of tadpole survival, hatching success, and mortality. (2) Hybrids from both reciprocal crosses show equal, and sometimes higher, measures of tadpole fitness than pure lineage crosses. Below we discuss these different findings in turn.

Differences in hatching success and survival between the two spring peeper lineages may in part be a by-product of neutral microevolutionary processes. For instance, within the Ontario contact zone, the Eastern lineage is the furthest from its presumed glacial refugium in the southern Appalachians (Austin et al. 2002, 2004) and protracted northerly migration after glacial retreat probably involved sequential founder events and genetic drift in populations with small effective size. Erosion of genetic variability through sequential founder effects during range expansion from glacial refugia and contemporary range fragmentation can combine to diminish population genetic diversity (Garner et al. 2004; Ficetola et al. 2007). In fact, the highest level of genetic diversity for spring peepers was found in populations closest to the posited refugia (Austin et al. 2002). Moreover, such genetic erosion is accentuated by inbreeding with consequent fitness reductions in other anurans (Hitchings and Beebee 1998). For example, genetic diversity affects hatching success and tadpole fitness in *Rana latastei* (Ficetola et al. 2007), and premetamorphic survival in *Bufo bufo* (Hitchings and Beebee 1998).

Alternatively, differential hatching success and tadpole survival among different environments may be a consequence of past selection. For example, habitat type and canopy cover have been shown to relate to thermal tolerance of larval amphibians, both among and within species (Freidenburg and Skelly 2004). Environmental temperature has also been shown to have important implications for geographical distribution, time of breeding, site and mode of egg deposition, egg and clutch size, and embryo and larval temperature tolerance, and development rate among frog species (Moore 1939; Volpe 1953; Pettus and Angleton 1967; Zweifel 1968; Licht 1971; Anderson 1972; Browning 1973), and even among conspecific populations (Moore 1949; Ruibal 1955; Volpe 1955; Brown 1967; Herreid and Kinney 1967).

Overall, much evidence to date demonstrates that both abiotic (temperature, UV-B radiation, hydroperiod, pH, salinity) and biotic (competition, predators, pathogens) factors can affect embryonic and tadpole development, even over relatively short evolutionary times (Skelly et al. 2002). It is not unreasonable then to anticipate that up to 5 million years of divergence (Stewart 2013) and geographical isolation in different paleoenvironments would have influenced phenotypes at various life-history stages, from ontogenetic trajectories shown here to previously demonstrated adult mating behavior (Stewart 2013). Early developmental or juvenile phases may even exaggerate evidence for local adaptations in life-history traits because of their decreased ability to disperse, migrate, and/or escape stressors compared with adults (Gomez-Mestre and Tejedo 2003; Vallin et al. 2013).

Our study also suggests that hybrid tadpoles between these two lineages have equal, and sometimes higher, values for fitness correlates when raised in isolation than either pure Eastern or pure Interior tadpoles (Figs. 2–4), despite some hybrid tadpoles exhibiting gross morphological and developmental deformities. Hybrid inviability evolves gradually and empirical evidence suggests that few recently diverged taxa have completely inviable F_1_ hybrids (Coyne and Orr 1989; Sasa et al. 1998; Presgraves 2002; Coyne and Orr 2004). For example, postzygotic isolation involving hybrid inviability is often incomplete until species have diverged for 2–3 million years for mammals (Prager and Wilson 1975), 10–20 million years for fish (Bolnick and Near 2005; Stelkens et al. 2010), and 20–30 million years for birds and frogs (Prager and Wilson 1975). Moreover, many genes responsible for intrinsic postzygotic isolation under the DMI model are partially recessive, and thus, the costs of hybridization may be manifested or exaggerated only in subsequent generations (e.g., as F_2_ or backcrossed descendents; Coyne and Orr 2004). Fitness correlates, as we measured them, show no evidence of hybrid inviability for F_1_ hybrids, but further investigations into F_2_ or backcrossed individuals is warranted.

We only investigated the consequences of hybridization on development from fertilization to metamorphosis and cannot speak to other potential aspects of postzygotic isolation mechanisms directly (e.g., hybrid mating success). Hybrid sterility, however, is suggested to evolve more quickly than hybrid inviability in many groups (Coyne and Orr 1997; Presgraves 2002; Coyne and Orr 2004) and may represent a postzygotic isolating barrier maintaining these lineage boundaries. For example, Sasa et al. (1998) found that among 116 anuran taxa surveyed, hybrid sterility evolved far quicker than inviability, and for considerations of the consequences of maladaptive hybridization such as reinforcement, may have equally strong fitness consequences (Muller 1940). Previous spring peeper work has shown significantly more nonmotile sperm in hybrids (Wang 2012) as well as mating behavioral dysfunction (Stewart 2013). Thus, the consequences to hybridization between these two lineages may become magnified later in life rather than simply reflecting DMI on early life-history traits. To accurately predict hybrid fitness, we need to understand the conditions under which hybrid genotypes survive and reproduce (Parris 2001). Thus, investigating postzygotic isolation may require the quantification of both intrinsic and extrinsic factors that impact hybrid fitness (Coyne and Orr 2004), many of which may operate at different evolutionary time scales.

Fitness consequences of anuran hybridization may also only be manifested under stressful environments, such as pathogenic infection (Parris 2004) or resource competition (Semlitsch 1993; Pfennig et al. 2007). Preliminary evidence from a pilot study (Supporting Information, Methods, Figure S1) suggests that spring peeper hybrid equality/superiority may be eliminated when raised in mixed rearing conditions (competition trials). Moreover, most hybrids raised with pure lineage individuals seem to not survive to metamorphosis, implying competitive inferiority. These preliminary competition trial results highlight the importance of inter- and intraspecific competition in wild assemblages, and recent research into developmental abnormalities has increasingly incorporated such perspectives, especially in anuran hybridization studies (Semlitsch 1993; Semlitsch et al. 1997; Parris 2001; Pfennig et al. 2007). Tadpole competition can cause longer development times, smaller mass at metamorphosis, reduced survival (Griffiths 1991; Blaustein and Margalit 1996), and even cannibalism (Pfennig et al. 1993) between frequently interacting individuals. All sampled populations within the Southwestern Ontario spring peeper contact zone contain both pure and hybrid individuals (Stewart 2013), raising the possibility of competition across all life-history stages.

Hybrid advantage may be evident as increased body size at metamorphosis, yet this may not ultimately represent any long-term benefit. For example, both size (Arendt 1997) and swimming speed (Walker et al. 2005) at the tadpole stage are effective antipredator adaptations but trade-offs have been noted (Arendt 2009). Tadpole speed confers an immediate benefit while size is only beneficial once large size is attained, suggesting that speed is a better antipredator strategy in the short term (Arendt 2009). If such a trade-off exists in spring peeper tadpoles, larger hybrid tadpoles may become more vulnerable to predation or competition through slower escape responses. Certainly, laboratory conditions themselves may contribute to underestimates of hybrid inviability, lacking important aspects of the natural context of wild populations (Kozak et al. 2012).

## Conclusion

Although ecological divergence is thought to be a common, and even pivotal initial step in the speciation process (Nosil 2012), reproductive isolation through the accumulation of genetic incompatibilities may precede any obvious ecological and/or morphological differentiation. Cryptic diversity between diverged intraspecific populations, especially in North America, has received little attention to date. Across the range of the spring peeper, some of the six deeply diverged lineages may well represent cryptic, fully reproductively isolated species with little demonstrable morphological distinction (Austin et al. 2004). Our study is the first to quantify the relative strength of postzygotic reproductive isolation on two comparatively newly diverged lineages of this species. Here, we show that hybrids between the Eastern and Interior spring peeper lineages exhibit equivalent, if not better, survivorship and growth when reared in isolation, raising interesting questions on the mechanisms maintaining the reduction in gene flow within this species. Although we do not demonstrate postzygotic isolation via hybrid inviability, extrinsic, context-dependent postzygotic isolation mechanisms may have equally strong, or possibly stronger, fitness consequences. Future studies should incorporate more ecologically relevant experimental conditions when assessing hybrid fitness.

## Supporting Information

**Data S1.** Methods

**Figure S1.** *Pseudacris crucifer* survival with competition.

**Table S1.** *Pseudacris crucifer* microsatellite primer information.
