# Supplementary material for: Testing for intraspecific postzygotic isolation between cryptic lineages of Pseudacris crucifer
Source: Ecol Evol. 2013 Oct 23;3(14):4621–30. doi: 10.1002/ece3.851 (PMC3867898; doi:10.1002/ece3.851)
Supplement: Supplementary file 1 [file ece30003-4621-SD1.docx]

**Supplementary Information**

**Methods**

Newly-hatched tadpoles not used for other experiments (and demonstrating no obvious deformities), were used for pilot competition trials. Here, groups of 5 full sibling tadpoles were paired with 5 other (full-sibling) tadpoles from either another family with the same lineage (pure or hybrid) or with 5 tadpoles from the opposite lineage (pure or hybrid) in a smaller (1 L) container. For these competition trials we had, in total, 2 containers with pure Interior and pure Eastern tadpoles, 1 container with pure Interior and Eastern hybrids, 1 container with pure Interior and Interior hybrids, 1 container with pure Eastern and Eastern hybrids, 1 container with pure Eastern and Interior hybrids, and 2 containers with Interior hybrids and Eastern hybrids, and 1 container each of tadpoles from similar genetic backgrounds but from different crosses (Eastern with Eastern, Interior with Interior, Eastern hybrid with Eastern hybrid, and Interior hybrid with Interior hybrid). In total we set up and followed 12 tadpole competition trials to metamorphosis. Rearing conditions were identical to those outlined in our previous experiments.

If tadpoles originated from a competition container comprised of tadpoles with the same haplotype (Interior with Interior hybrids, or Eastern with Eastern hybrids) we further distinguished individuals using 5 polymorphic (one previously published; Degner et al. 2009) microsatellites, in triplex (Pcru32, Prcu 10, Pcru 05) and duplex (Pcru 11, Pcru 21) reactions (see Supplementary Information, Table S1 for primer quantities) following protocol outlined in Stewart (2013).

For our competition trials we assessed survival rate to metamorphosis (genotyping individuals within mixed containers as stated above). Small sample sizes precluded the use of formal statistical tests for competition trials and we use descriptive measures only.

*Results from Tadpole Competition Trials*

We found that tadpoles raised in mixed family groups exhibited higher mortalities compared to those raised in isolation. Pure Interior and pure Eastern tadpoles raised together had 40% overall mortality, most of which were pure Eastern tadpoles. Hybrids with Interior haplotypes had 100% mortality when raised with pure Interior tadpoles but 100% survival when raised with pure Eastern tadpoles. Similarly, hybrids with Eastern haplotypes showed 100% mortality when raised with pure Eastern tadpoles and only 40% survival when raised with pure Interior tadpoles. Hybrids with Eastern haplotypes also showed 100% mortality when raised with other hybrids with Interior haplotypes, which consequently showed 100% survival (see Figure S1).

**Table S1.** ***Pseudacris crucifer* microsatellite primer information.** Primer ID and GenBank Accession numbers, author affiliation, allele ranges, and PCR reaction forward and reverse primer quantities for 5 microsatellite markers used during the genotyping of *P. crucifer*.

| Primer ID | Forward Sequence (5’ to 3’) | Reverse Sequence (3’ to 5’) | T_a_ | GenBank Accession # | Reference | Allele Range | Repeat Motif | Quantity PCR Reaction Primer |
| --- | --- | --- | --- | --- | --- | --- | --- | --- |
| Pcru32 | CCCTACATAGGATTG | GTACACCCACCAAAAGTGCT | 54 | EF190904 | Van Buskirk,et al. (2006) unpubl. | 121-163 | (CA)_11_ | 0.2 µl |
| Pcru10 | GGGGGATGCAGAATT | CGGTTCCTATTGAAGAACA | 54 | EF190896 | Degner et al. (2009) | 187-213 | (CA)_13_ | 0.5 µl |
| Pcru05 | CATTTATAAGCAGTGCAGAGAGG | TGCATTGATGTTTCTCATGG | 54 | EF190892 | Van Buskirk,et al. (2006) unpubl. | 320-366 | (CA)_13_ | 0.5 µl |
| Pcru11 | GGATATGCTCACATG | CCCAGATTCCAAGTGTTTTC | 55 | EF190897 | Van Buskirk,et al. (2006) unpubl. | 112-168 | (GT)_16_ | 0.3 µl |
| Pcru21 | TGGAGACATCATTGC | CCCTTGGTCCTGAATAGGTT | 55 | EF190900 | Van Buskirk,et al. (2006) unpubl. | 210-264 | (CA)_14_ | 0.45 µl |

**Figure S1. *Pseudacris crucifer* survival with competition.** *Pseudacris crucifer* tadpole average survival (%) for Pure Interior (I), Pure Eastern (E), Hybrids with Interior haplotypes (HI), and Hybrids with Eastern haplotypes (I, E, HI, HE, respectively) when raised in mixed groups. Treatments (mixed rearing groups) denoted on X-axis.


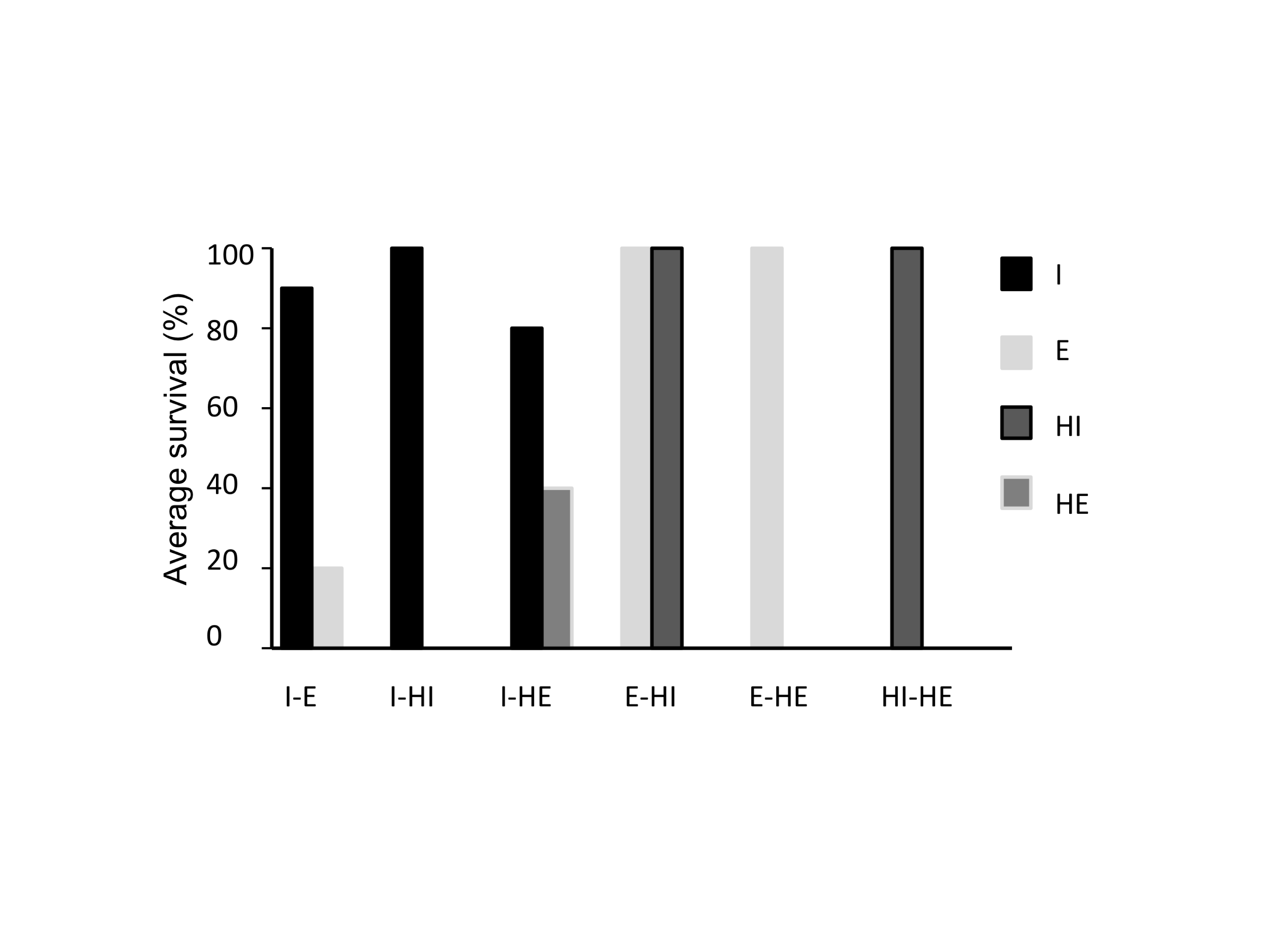


**References**

Degner JF, Hether TD, Hoffman EA (2009) Eight novel tetranucleotide and five cross-species dinucleotide microsatellite loci for ornate chorus frog (*Pseudacris ornata*). Mol. Ecol. Res. 9: 622-624.
